# Epigenetic age acceleration predicts cancer, cardiovascular, and all-cause mortality in a German case cohort

**DOI:** 10.1186/s13148-016-0228-z

**Published:** 2016-06-03

**Authors:** Laura Perna, Yan Zhang, Ute Mons, Bernd Holleczek, Kai-Uwe Saum, Hermann Brenner

**Affiliations:** Division of Clinical Epidemiology and Aging Research, German Cancer Research Center (DKFZ), Im Neuenheimer Feld 581/TP4, 69120 Heidelberg, Germany; Saarland Cancer Registry, Präsident Baltz Straße 5, 66119 Saarbrücken, Germany; Network Aging Research (NAR), University of Heidelberg, Bergheimer Straße 20, 69115 Heidelberg, Germany

**Keywords:** DNA methylation age, Epigenetic clock, Epigenetic age acceleration, Mortality risk

## Abstract

**Background:**

Previous studies have developed models predicting methylation age from DNA methylation in blood and other tissues (epigenetic clock) and suggested the difference between DNA methylation and chronological ages as a marker of healthy aging. The goal of this study was to confirm and expand such observations by investigating whether different concepts of the epigenetic clocks in a population-based cohort are associated with cancer, cardiovascular, and all-cause mortality.

**Results:**

DNA methylation age was estimated in a cohort of 1863 older people, and the difference between age predicted by DNA methylation and chronological age (Δ_age_) was calculated. A case-cohort design and weighted proportional Cox hazard models were used to estimate associations of Δ_age_ with cancer, cardiovascular, and all-cause mortality. Hazard ratios for Δ_age_ (per 5 years) calculated using the epigenetic clock developed by Horvath were 1.23 (95 % CI 1.10–1.38) for all-cause mortality, 1.22 (95 % CI 1.03–1.45) for cancer mortality, and 1.19 (95 % CI 0.98–1.43) for cardiovascular mortality after adjustment for batch effects, age, sex, educational level, history of chronic diseases, hypertension, smoking status, body mass index, and leucocyte distribution. Associations were similar but weaker for Δ_age_ calculated using the epigenetic clock developed by Hannum.

**Conclusions:**

These results show that age acceleration in terms of the difference between age predicted by DNA methylation and chronological age is an independent predictor of all-cause and cause-specific mortality and may be useful as a general marker of healthy aging.

**Electronic supplementary material:**

The online version of this article (doi:10.1186/s13148-016-0228-z) contains supplementary material, which is available to authorized users.

## Background

DNA methylation is a form of epigenetic control involved in several cellular processes and in the regulation of gene expression, especially through gene silencing [1]. Modifications of DNA methylation are influenced by genetic and environmental factors, including age, a phenomenon called “epigenetic drift” [2]. Such age-related modifications are bidirectional (they both show an increase and a decrease in methylation, depending on the sites), vary between individuals of the same age, and differ across the genome [3].

Several models, also called “epigenetic clock,” have been developed, predicting chronological age from DNA methylation (DNAm_age_) with high accuracy [4, 5]. An Italian study showed that semi-supercentenarians had a lower epigenetic age than age-matched controls and that their offspring’s epigenetic age was systematically lower than their chronological age [6], suggesting that epigenetic processes play a role in healthy aging. An increasing number of studies indicate that the discrepancy between DNAm_age_ and chronological age may be associated with age-related diseases and mortality. A few studies indicated that epigenetic age acceleration (i.e., predicted methylation age exceeding chronological age) was significantly associated with increased mortality risk [7–9], frailty [10], and lower cognitive performance [11], including neuropathological markers of Alzheimer’s disease [12]. Age acceleration was also observed in brain tissues of people affected by Trisomy 21, who have a well-known increased risk of developing Alzheimer’s disease [13] and there is emerging evidence that blood epigenetic age may predict cancer incidence [14] and mortality [15].

This literature seems to suggest that age acceleration in terms of the difference between age predicted by DNA methylation and chronological age could be a useful marker of healthy aging. However, in order to corroborate such findings, it is of importance to replicate the above results in different population-based cohorts and, additionally, to explore whether epigenetic age acceleration is also associated with other relevant age-related outcomes, such as cardiovascular disease (CVD) mortality.

We aimed to investigate whether the discrepancy between DNAm_age_ as predicted by the models of Hannum et al. [4] (hereinafter also DNAm_age (Hannum)_) and Horvath [5] (hereinafter also DNAm_age (Horvath)_) and chronological age is associated with cancer, cardiovascular, and all-cause mortality in a population-based cohort of older people.

## Methods

### Study population and study design

Measurements of DNA methylation in blood were performed in a baseline subsample of the ESTHER cohort, which is a large population-based epidemiological study conducted in the German State of Saarland with the aim of assessing chances of prevention and early detection of chronic diseases. As described in more detail in Saum et al. [16], between July 2000 and December 2002, general practitioners recruited 9949 older adults aged 50–75 years during a regular health check-up.

Socio-demographic, lifestyle, and health characteristics were collected using standardized self-administered questionnaires. Self-reported diagnoses of prevalent cardiovascular diseases, cancer, hypertension, and diabetes mellitus were validated through medical records. Information on survival status was obtained by record linkage with population registries. Cause of death was ascertained according to death certificate. All participants provided blood samples, which were stored at −80 °C. The Ethics Committee of the Medical Faculty of the University of Heidelberg and of the Physician Board of Saarland approved the study and participation was conditional on written informed consent.

Due to financial constraints, DNA was extracted only from a subsample of 3499 ESTHER participants who were recruited in the initial phase of the enrolment between July 2000 and March 2001 and who therefore had the longest follow-up time. Among these, epigenetic measurements were conducted in a randomly selected subcohort of 1548 participants irrespective of survival status as well as in all participants who had died during follow-up (cases) and did not overlap with the randomly selected subcohort (*N* = 316). In total, 1864 participants with epigenetic measurements were therefore available for the present case-cohort study.

### DNA methylation measurement

Blood DNA was extracted using a salting out procedure [17] and measured using PicoGreen (Invitrogen, Darmstadt, Germany). DNA methylation profiles were measured using the Infinium HumanMethylation450 BeadChip (Illumina Inc.) in the Genomics and Proteomics Core Facility at the German Cancer Research Center, Heidelberg. As quality control, three random DNA samples for each plate were used as duplicates for replication. Methylation levels at each CpG were calculated with Illumina’s Genomestudio 2011.1, Modul M Version 1.9.0 as previously described in detail [18].

### Prediction of DNAm_age_

DNAm_age (Horvath)_ was calculated using the R tutorial of the pertinent publication [5] and DNAm_age (Hannum)_ as the sum of the beta values multiplied by the respective regression coefficients reported by Hannum et al. [4] as already described by Marioni et al. [7]. For all calculations of DNAm_age_, we only used signals of probes with a detection *p* value ≤0.05.

### Statistical analysis

We used descriptive statistics to show the distribution of socio-demographic variables and prevalent diseases at baseline separately among the participants of the randomly selected subcohort who survived over the follow-up and among the cases. We also differentiated the cases between those participants of the subcohort who deceased during the follow-up (cases within the subcohort) and those who were specifically selected among deceased participants (cases outside the subcohort).

Following Marioni et al. [7], we calculated the difference between age predicted by DNA methylation and chronological age (Δ_age_; epigenetic age acceleration) and estimated hazard ratios (HR) for the association of Δ_age_ (per 5 years of age acceleration) with all-cause and cause-specific mortality. HRs were estimated using the SAS® program code implementing weighted proportional Cox hazard models as illustrated in detail by Kulathinal and colleagues [19]. Effect estimates were adjusted in model 1 for batch effects, chronological age (continuous), sex, and leucocyte distribution including the following cell types: CD56 natural killer cells, CD14 monocytes, CD4 T-cells, CD8 T-cells, CD19 B-cells, and granulocytes. Proportions of leucocyte cells were estimated from DNA methylation using the algorithm from Houseman et al. [20]. Model 2 was additionally adjusted for educational level, history of cancer, history of CVD, hypertension, diabetes mellitus, smoking status, and body mass index. Sensitivity analyses were conducted without adding leucocyte distribution as a covariate in model 1.

Scatter plots were generated in order to illustrate the relationship between chronological age and DNAm_age_. Pearson correlation coefficient (*r*) between chronological and methylation age were calculated both for DNAm_age (Horvath)_ and DNAm_age (Hannum)_.

In order to explore specific biological components of the epigenetic clock associated with mortality, we estimated, in Cox models adjusted for chronological age, sex, leucocyte distribution, and batch effects, which of the 353 CpGs of Horvath’s epigenetic clock were significantly associated with all-cause mortality. Multiple testing adjustments were performed both by controlling the false discovery rate (FDR) and using the Bonferroni correction.

With the exception of DNAm_age (Horvath)_, which we calculated using R software version 3.1.1, we conducted all analyses using SAS® statistical software version 9.4 (SAS® Institute Inc., Cary, NC, USA).

## Results

The subsample of the ESTHER cohort consisted of 1863 participants with 1260 (67.6 %) survivors and 602 (32.3 %) cases (316 selected cases and 286 deceased participants within the subcohort) and 1 participant with missing information relating to survival status (Table 1). Overall, we recorded 235 (12.6 %) deaths due to cancer (*N* = 95 among women and *N* = 140 among men) and 194 (10.4 %) cardiovascular deaths (*N* = 80 among women and *N* = 114 among men). The majority of deceased participants were men both among cases outside the subcohort (56.3 %) and within the subcohort (60.5 %). Cases showed more age-related diseases at baseline, such as CVD, hypertension, and diabetes mellitus, than survivors. Also, there were more current smokers among cases within (27.0 %) and outside (24.3 %) the subcohort than among surviving participants (16.4 %).

**Table 1:** Distribution of socio-demographic variables and prevalent diseases at baseline among participants of the case cohort (ESTHER study 2000–2001)

Variable	Survivors	Cases within the subcohort^a^	Cases outside the subcohort^b^
Sex			
Female	721 (57.2)	113 (39.5)	138 (43.7)
Men	539 (42.8)	173 (60.5)	178 (56.3)
Age			
50–65	905 (71.8)	123 (43.0)	163 (51.6)
66–75	355 (28.2)	163 (57.0)	153 (48.4)
Years of school education			
>9 years	334 (27.0)	56 (20.4)	63 (20.5)
≤9 years	902 (73.0)	218 (80.6)	244 (79.5)
History of cancer disease			
No	1201 (95.3)	254 (88.8)	268 (84.8)
Yes	59 (4.7)	32 (11.1)	48 (15.2)
History of cardiovascular disease			
No	1048 (83.2)	171 (59.8)	206 (65.2)
Yes	212 (16.8)	115 (40.2)	110 (34.8)
Hypertension			
No	714 (56.7)	142 (50.5)	154 (49.2)
Yes	545 (43.3)	139 (49.5)	159 (50.8)
Diabetes mellitus			
No	1068 (85.9)	216 (76.1)	227 (73.0)
Yes	176 (14.2)	68 (23.9)	84 (27.0)
Body mass index (kg/m^2^)			
<25	341 (27.1)	79 (27.6)	97 (30.7)
25–<30	594 (47.1)	124 (43.4)	132 (41.8)
≥30	325 (25.8)	83 (29.0)	87 (27.5)
Smoking			
Never/former	1034 (83.6)	203 (73.0)	233 (75.7)
Current	203 (16.4)	75 (27.0)	75 (24.3)

^a^Participants of the randomly selected subcohort who were deceased during the follow-up (2000–2013)

^b^Participants specifically selected among deceased participants

**Fig. 1:**
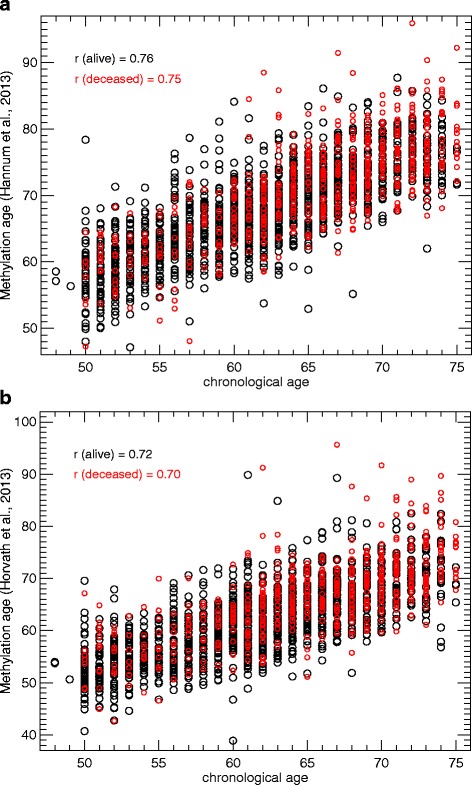
**a** Relationship between DNA methylation age estimated with Hannum’s predictor and chronological age. **b** Relationship between DNA methylation age estimated with Horvath’s predictor and chronological age

Mean chronological age at baseline in the total study population was 62.5 (SD 6.6; min 48.0, max 75.0). Mean of DNAm_age (Horvath)_ was 63.0 (SD = 7.5; min = 38.9 max 95.7) and mean of DNAm_age (Hannum)_ was 68.6 (SD = 7.1; min = 47.1 max 95.9). Pearson correlation coefficient between chronological and methylation age was 0.73 (*p* < 0.0001) for DNAm_age (Horvath)_ and 0.77 (*p* < 0.0001) for DNAm_age (Hannum)_. Panels a and b of Fig. 1 display scatter plots showing the relationship between chronological and DNAm_age_ both among deceased participants (red circles) and those alive at last follow-up (black circles). Also, there was a strong linear relationship between DNAm_age (Horvath)_ and DNAm_age (Hannum)_ (*r* = 0.80; *p* < 0.0001).

Weighted proportional Cox hazard models revealed that irrespective of which predictor was used, a DNA methylation age higher than chronological age was associated with higher mortality. In Cox models adjusted for age, sex, batch effects, and leucocyte distribution HRs for the association of Δ_age_ (per 5 years) with all-cause mortality showed a statistically significant increase in mortality by 22 % using DNAm_age (Horvath)_ and an increase in mortality by 16 % using DNAm_age (Hannum)_ (Table 2). In the fully adjusted models, HR estimates for Δ_age_ were stronger for DNAm_age (Horvath)_ (HR = 1.23; 95 % CI 1.10–1.38) than for DNAm_age (Hannum)_, which were no longer statistically significant (HR = 1.10; 95 % CI 0.94–1.29).

**Table 2:** Associations of differences between Δ_age_ (per 5 years) according to different predictors of DNAm_age_ with all-cause and cause-specific mortality

Causes of death (*N* of events; %)	Predictor	Cox model 1^a^ (Hazard ratios)	Cox model 2^b^ (Hazard ratios)
All-cause mortality			
(602; 32.3)	DNAm_age (Horvath)_	1.22 (1.10–1.36)	1.23 (1.10–1.38)
(602; 32.3)	DNAm_age (Hannum)_	1.16 (1.00–1.34)*	1.10 (0.94–1.29)
Cancer mortality			
(235; 12.6)	DNAm_age (Horvath)_	1.20 (1.03–1.39)	1.22 (1.03–1.45)
(235; 12.6)	DNAm_age (Hannum)_	1.08 (0.87–1.35)	1.03 (0.80–1.33)
Cardiovascular disease mortality			
(194; 10.4)	DNAm_age (Horvath)_	1.20 (1.02–1.42)	1.19 (0.98–1.43)
(194; 10.4)	DNAm_age (Hannum)_	1.13 (0.89–1.44)	1.00 (0.79–1.29)

*p = 0.0408

^a^Model 1: adjusted for chronological age (continuous), sex, batch effects, and leucocyte distribution

^b^Model 2: additionally adjusted for educational level, history of cancer diseases, history of CVD, hypertension, diabetes mellitus, smoking status (never/former vs. current), and body mass index (continuous)

Epigenetic age acceleration was also associated with higher cancer and CVD mortality in Cox models using Horvath’s predictor and adjusted for age, sex, batch effects, and leucocyte distribution (HR = 1.20; 95 % CI 1.03–1.39 and HR = 1.20; 95 % CI 1.02–1.42, respectively). Cox models for DNAm_age (Hannum)_ also showed an increased mortality both for cancer and CVD mortality in partially adjusted models but estimates were weaker and they were not statistically significant (HR = 1.08; 95 % CI 0.87–1.35 and HR = 1.13; 95 % CI 0.89–1.44, respectively).

Associations of epigenetic age acceleration with all-cause and cause-specific mortality were very similar among women and men (Table 3).

**Table 3:** Associations of differences between Δ_age_ (per 5 years) according to different predictors of DNAm_age_ with all-cause and cause-specific mortality by sex

	Number of events; %	Predictor	Cox model 1^a^ (Hazard ratios)	Cox model 1^b^ (Hazard ratios)
All-cause mortality				
Women	251; 41.7	DNAm_age (Horvath)_	1.27 (1.07–1.52)	1.24 (1.02–1.52)
		DNAm_age (Hannum)_	1.19 (0.94–1.51)	1.05 (0.82–1.36)
Men	351; 58.3	DNAm_age (Horvath)_	1.23 (1.05–1.43)	1.28 (1.09–1.51)
		DNAm_age (Hannum)_	1.14 (0.94–1.40)	1.14 (0.92–1.41)
Cancer mortality				
Women	95; 40.4	DNAm_age (Horvath)_	1.20 (0.93–1.54)	1.21 (0.88–1.65)
		DNAm_age (Hannum)_	1.02 (0.69–1.50)	0.89 (0.58–1.36)
Men	140; 59.6	DNAm_age (Horvath)_	1.18 (0.95–1.47)	1.25 (0.98–1.59)
		DNAm_age (Hannum)_	1.08 (0.80–1.46)	1.12 (0.79–1.58)
CVD mortality				
Women	80; 41.2	DNAm_age (Horvath)_	1.17 (0.91–1.51)	1.13 (0.82–1.55)
		DNAm_age (Hannum)_	1.21 (0.85–1.72)	1.01 (0.64–1.61)
Men	114; 58.8	DNAm_age (Horvath)_	1.25 (0.98–1.59)	1.29 (0.99–1.68)
		DNAm_age (Hannum)_	1.06 (0.77–1.47)	1.00 (0.71–1.42)

*CVD* cardiovascular disease

^a^Model 1: adjusted for chronological age (continuous), sex, batch effects, and leucocyte distribution

^b^Model 2: additionally adjusted for educational level, history of cancer diseases, history of CVD, hypertension, diabetes mellitus, smoking status (never/former vs. current), and Body Mass Index (continuous)

Sensitivity analyses conducted without adding leucocyte distribution as covariate in model 1 revealed weaker point estimates for DNAm_age (Horvath)_ (HR_(all-cause mortality)_ = 1.17; 95 % CI 1.06–1.30; HR _(cancer mortality)_ = 1.15; 95 % CI 0.99–1.34; HR_(CVD mortality)_ = 1.15; 95 % CI 0.98–1.34) and stronger estimates for DNAm_age (Hannum)_ (HR_(all-cause mortality)_ = 1.21; 95 % CI 1.06–1.38; HR _(cancer mortality)_ = 1.11; 95 % CI 0.91–1.37; HR_(CVD mortality)_ = 1.20; 95 % CI 0.96–1.50) .

Cox regression analyses for the 353 CpGs of Horvath’s epigenetic clock revealed that 6 CpGs (cg19724470 in *CD27*; cg15804973 in *MAP3K5*, cg25564800 in *KPNA1*; cg01820374 in *LAG3*; cg01511567 in *SSRP1*; cg26614073 in *SCAP*) showed a statistically significant association with mortality after FDR adjustment and 3 CpGs after Bonferroni correction (Additional file 1: Table S1). Without adjustment for multiple testing, we found that 33 CpGs were associated with mortality.

## Discussion

In this cohort of older adults, epigenetic age acceleration in terms of the difference between methylation age and chronological age was associated with increased total, cancer, and CVD mortality even after adjustment for a large number of potential confounders. These findings strengthen suggestions that epigenetic age acceleration may be a general (bio-)marker of healthy aging.

Being the first study addressing CVD mortality, we cannot compare our results relating to such outcome with other studies, but our findings relating to all-cause mortality were quite comparable with those from a recent publication. In particular, Marioni and colleagues [7] found that Δ_age_ (per 5 years) was significantly associated with a 21 % greater mortality risk in a model adjusted for chronological age and sex using DNAm_age (Hannum)_ (HR = 1.21; 95 % CI 1.14–1.29) and with a 11 % increase using DNAm_age (Horvath)_ (1.11; 95 % CI 1.05–1.18). The consistent results obtained for all-cause mortality reinforce suggestions that epigenetic age acceleration may be regarded as a possible candidate biomarker of healthy aging.

In our main analysis with adjustment for leucocyte distribution, the association between Δ_age_ and mortality was stronger for DNAm_age (Horvath)_ than for DNAm_age (Hannum)_. In sensitivity analyses without adjustment for leucocyte distribution, point estimates for DNAm_age (Hannum)_, but not for DNAm_age (Horvath)_, were stronger than the corresponding point estimates adjusted for leukocyte distribution. This seems to suggest that Horvath’s predictor, being developed from several human tissues and cell types and not only from blood, might be more robust than the blood-based predictor from Hannum and also less sensitive to changes in blood cell composition, which could mediate the association between Δ_age_ and mortality [7]. However, given that the direction of the association remained stable and that there was only a moderate change in the strength of the association for both predictors, we can infer that leucocyte distribution did not have a major influence on the association between epigenetic clocks and mortality in our cohort.

Mean DNAm_age_ predicted by the Hannum’s predictor was approximately 6 years higher than mean chronological age, an overestimation also observed in the cohorts analyzed by Marioni and colleagues [7]. Given the very good correlation between methylation and chronological age and the strong linear relationship between DNAm_age (Horvath)_ and DNAm_age (Hannum)_, we did not deem it necessary to account for the overestimation.

Our findings indicating associations between Δ_age_ and cancer mortality are in line with studies showing that methylation changes might represent an early event in the development of cancerous cells [2, 3] and be able to predict cancer incidence [14, 15]. Our results suggest that epigenetic age acceleration might also be an indicator for a more aggressive course of tumor disease with increased risk of a cancer-related fatal event, which supports the results of Zheng et al. [15] who also found that the discrepancy between epigenetic and chronological ages predicted cancer mortality.

Our findings revealing that epigenetic age acceleration was also associated with a 20 % greater CVD mortality risk (for estimates based on Horvath’s predictor) reveal a magnitude of association comparable to the observed association related to cancer mortality, even if CVD mortality was less frequent than cancer mortality (194 vs 235, respectively) and therefore CVD mortality models had less statistical power.

A possible explanation for the observed similar magnitude of association is that, similar to cancer, the development of CVD also involves focal proliferative events [3, 21] and this might point to a relationship between aberrant methylation in older age and focal proliferation processes. In particular, on the one hand, an abnormal epigenetic drift might lead to an aggravation of focal proliferative events leading, *inter alia*, to cardiovascular and tumor diseases, and on the other hand, focal proliferative events might lead to further abnormal epigenetic drift [3] with exacerbation of the cardiovascular and cancer pathologies leading eventually to higher mortality. The design of our study with epigenetic measurements obtained several years prior to the event supports a role of epigenetic changes leading to the exacerbation of cancer and CVD disease, but it cannot definitively exclude reciprocal influences, as the event leading to greater mortality might have already affected the epigenetic status at an early stage of the disease and be reflected in the epigenetic measurements.

Lin and colleagues [9] tested associations of single CpG-derived age predictions with mortality in the LBC1936 cohort. Two CpGs that showed significant results before adjustment for multiple testing were overlapping with our findings (cg19724470 and cg15804973). Further studies should explore biological properties of such genes and the possible pathways leading to early mortality.

Strengths of our study were analyses conducted with two different predictors, the large and representative sample size, and adjustment for important confounders; main limitations were the lack of differential blood counts and the impossibility to ascertain in our population the exact temporal relationship between epigenetic age acceleration and focal proliferative events.

## Conclusions

In conclusion, our study further confirms the validity of estimates of DNAm_age_ based on Horvath’s and Hannum’s epigenetic clocks, it expands previous observations by including CVD mortality, and it indicates that epigenetic age acceleration might be both a useful marker of healthy aging among older people as well as a prognostic marker.

## Additional file

Additional file 1: Table S1. Associations of single CpG sites with all-cause mortality. (XLSX 12 kb)
